# Novel Therapeutics for Anthracycline Induced Cardiotoxicity

**DOI:** 10.3389/fcvm.2022.863314

**Published:** 2022-04-22

**Authors:** Jacqueline T. Vuong, Ashley F. Stein-Merlob, Richard K. Cheng, Eric H. Yang

**Affiliations:** ^1^Department of Medicine, Ronald Reagan UCLA Medical Center, Los Angeles, CA, United States; ^2^Division of Cardiology, Department of Medicine, Ronald Reagan UCLA Medical Center, Los Angeles, CA, United States; ^3^Division of Cardiology, Department of Medicine, University of Washington, Seattle, WA, United States; ^4^UCLA Cardio-Oncology Program, Division of Cardiology, Department of Medicine, University of California, Los Angeles, Los Angeles, CA, United States

**Keywords:** cardio-oncology, anthracyclines, cardiotoxicity, cardiomyopathy, heart failure

## Abstract

Anthracyclines remain an essential component of the treatment of many hematologic and solid organ malignancies, but has important implications on cardiovascular disease. Anthracycline induced cardiotoxicity (AIC) ranges from asymptomatic LV dysfunction to highly morbid end- stage heart failure. As cancer survivorship improves, the detection and treatment of AIC becomes more crucial to improve patient outcomes. Current treatment modalities for AIC have been largely extrapolated from treatment of conventional heart failure, but developing effective therapies specific to AIC is an area of growing research interest. This review summarizes the current evidence behind the use of neurohormonal agents, dexrazoxane, and resynchronization therapy in AIC, evaluates the clinical outcomes of advanced therapy and heart transplantation in AIC, and explores future horizons for treatment utilizing gene therapy, stem cell therapy, and mechanism-specific targets.

## Introduction

**FIGURE 1:**
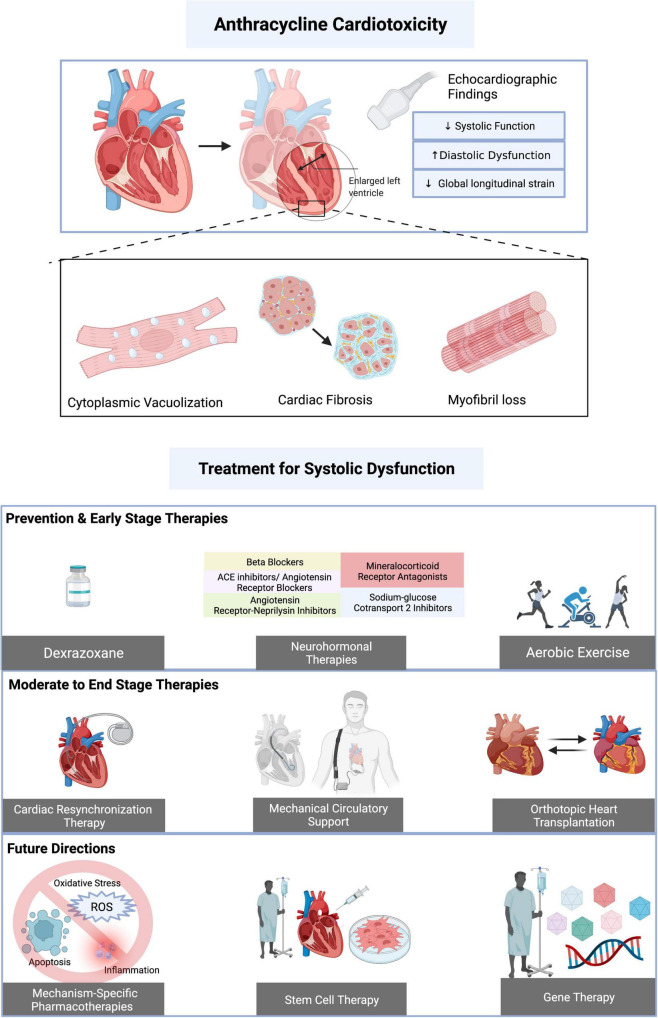
Central illustration. Summary of anthracycline induced cardiotoxicity (AIC) and treatment options. AIC on a cellular level is mediated by cytoplasmic vacuolization, cardiac fibrosis, and myofibril loss and is associated with echocardiographic of decreased systolic function, increased diastolic dysfunction, and decreased global longitudinal strain. Potentially preventative and/or investigational therapies for AIC associated systolic dysfunction include dexrazoxane, neurohormonal pharmacologic therapy, and aerobic exercise. Moderate to end stage therapy considerations include cardiac resynchronization therapy, mechanical circulatory support, and orthotopic heart transplantation. Therapies such as stem cell therapy, gene therapy, and targeting of AIC-specific mechanisms (such as apoptosis, reactive oxygen species production, and inflammation) are under ongoing investigation. Created with BioRender.com.

Despite many recent advances in cancer treatments, anthracycline therapies remain an essential component in the successful treatment of multiple hematologic and solid organ malignancies. As cancer survivorship improves, increased efforts have been made to understand and mitigate the short- and long-term toxicities of chemotherapies. Of particular concern for anthracyclines is the development of highly morbid anthracycline-induced cardiotoxicity (AIC), where manifestations can range from asymptomatic electrocardiogram (ECG) changes and left ventricular (LV) dysfunction to profound cardiomyopathy and end-stage heart failure (HF). This narrative review aims to discuss the interplay of proposed mechanisms of anthracycline cardiotoxicity and contemporary evidence for pharmacologic, advanced, and investigational therapies in the prevention and treatment of AIC (Central Illustration, Figure 1).

## Overview of Mechanisms of Cardiotoxicity With Pharmacologic Targets

**FIGURE 2:**
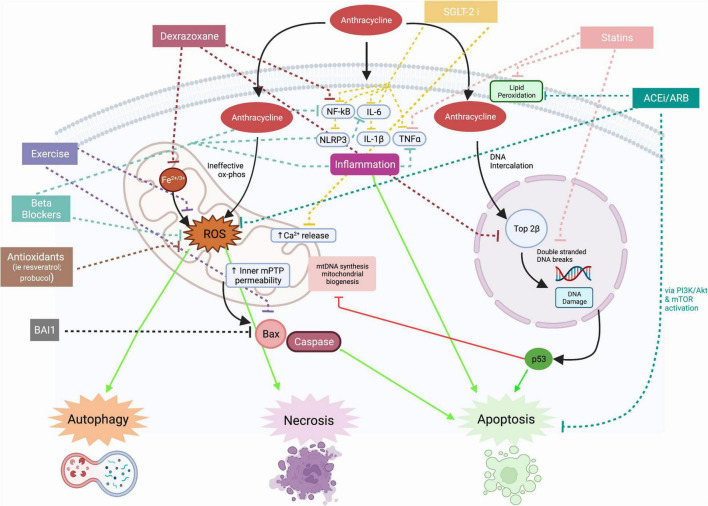
Mechanisms of anthracycline cardiotoxicity and effects of therapies. Mitochondrial effects of anthracycline induced cardiotoxicity include production of reactive oxygen species, calcium dysregulation, impaired mitochondrial biogenesis, and disruption in mitochondrial membrane integrity, leading to release of apoptotic molecules such as bcl-2-associated X protein (Bax). The effects of anthracycline induced cardiotoxicity on nuclei include DNA intercalation and binding to Topoisomerase 2β to cause double stranded DNA breaks. DNA damage releases pro-apoptotic factors such as p53. Anthracyclines increase the expression of pro-inflammatory cytokines such as NF-kB, IL-6, NLRP3, IL-1β, and TNF-α. Proposed therapies have inhibitory effects on inflammation, reactive oxygen species production, DNA damage and apoptosis. Solid lines indicate mechanisms of anthracycline cardiotoxicity and dotted lines indicate mechanisms of proposed therapies. ACEi, angiotensin converting enzyme inhibitor; ARB, angiotensin II receptor blocker; BAI1, BAX activation inhibitor 1; Bax, bcl-2-associated X protein; Ca, calcium; Fe2+/3+, iron; IL, interleukin; mPTP, mitochondrial permeability transition pore; mtDNA, mitochondrial DNA; NF-kB, nuclear factor kappa B; NLRP3, NLR family pyrin domain containing 3; ox phos, oxidative phosphorylation; ROS, reactive oxygen species; TNFα, tumor necrosis factor alpha; Top2β, topoisomerase 2β; SGLT2i, sodium glucose cotransporter 2 inhibitor; PI3K, phosphinositide 3-kinase; Akt, protein kinase B; mTOR, mammalian target of rapamycin. Created with BioRender.com.

To understand the current and investigational pharmacologic targets, an understanding of the mechanisms of AIC is essential. Mechanisms of anthracycline cardiotoxicity are multifactorial and involve pathways in DNA damage, mitochondrial dysfunction, oxidative stress, inflammation, and apoptosis promotion (Figure 2). Cardiac samples obtained from autopsy of patients with AIC demonstrate necrotic cells within the ventricular wall, interstitial fibrosis, cytoplasmic vacuolization, and marked reduction in the number of cardiomyocytes and myofibrils (1, 2). As anthracyclines preferentially accumulate in mitochondria and nuclei, the increased mitochondrial density and high energy demands of cardiomyocytes may explain the predilection for cardiotoxicity.

Anthracyclines interfere with many mitochondrial respiratory chain complexes involved in oxidative phosphorylation, leading to ineffective redox reactions and the formation of reactive oxygen species (ROS) (3). ROS production is exacerbated in the presence of iron and ROS interaction with various membrane and mitochondrial DNA constituents leads to alterations in autophagy and promotion of cardiomyocyte apoptosis and necrosis (4). Disruption of the integrity of mitochondrial membranes leads to release of pro-apoptotic factors (3). The depletion of cellular ATP and promotion of inner mitochondrial membrane permeability transition pore opening (mPTP) has also been associated with increased necrotic cardiomyocyte death (5). Anthracyclines also activate pro-inflammatory pathways involving nuclear factor-kB (NF-kB) and tumor necrosis factor alpha (TNF-α) and upregulate the transcription of NLRP3, interleukin (IL)-1β and IL-6, key inflammatory mediators of heart failure pathogenesis (6). Cardiomyocyte death leads to further activation of inflammatory cascades and ROS production, leading to functional and structural changes in the myocardium that is marked by fibrosis and electrical alterations (7, 8).

In addition to oxidative stress and inflammation, DNA intercalation by anthracyclines contributes to cardiotoxicity. Anthracycline binding to topoisomerase 2 beta causes double-stranded DNA breaks and inhibits transcription of several regulators of cardiac metabolism, leading to defective mitochondrial biogenesis and function and thereby indirectly contributing to exacerbation of ROS production (9). The DNA and nuclear damage leads to p53 activation and activation of pro-apoptotic pathways (9). As such, levels of pro-apoptotic molecules, such as Bax and caspase-3, have been found to be upregulated in rat hearts treated with anthracyclines (10).

## Pharmacologic Prevention of Anthracycline Induced Cardiotoxicity

Increasing recognition of the significant morbidity and mortality associated with AIC has led to exploration of treatment modalities to prevent the development of AIC. In preclinical studies, significant acute cardiotoxicity occurs at the time of the initial administration of anthracyclines that starts a cascade leading to the eventual development of LV dysfunction and HF. The most well-studied therapies include conventional heart failure therapies, including angiotensin converting enzyme (ACE) inhibitors and beta blockers, and dexrazoxane; additionally, there are multiple investigational treatments currently undergoing evaluation. The preclinical studies for the various therapies mentioned below are summarized in Table 1, while clinical studies are summarized in Table 2.

**TABLE 1:** Description of example preclinical studies and summary of therapeutic effect on AIC parameters.

Study	Study Design	Therapies	Findings (compared to anthracycline + no therapy)					
			
			LV systolic function	LV dimension	Fibrosis	ROS	Apoptosis	Other
**ACEi/ARB**								
Hullin et al. (25)	Mouse model Acute DOX (×1)	Enalapril	↔	↔	NA	NA	NA	
	Mouse model Chronic DOX (weekly ×5)	Enalapril	↑	↓	NA	NA	NA	↑ Activation PI3K/AKT/mTOR
Hiona et al. (12)	Rat model Chronic DOX (weekly ×6)	Enalapril	↑	NA	NA	↓	↔	↑ Mitochondrial function
								↑ %Fractional shortening
Abd El-Aziz et al. (121)	Rat model Acute DOX (×1)	Captopril or enalapril	NA	NA	NA	↓	NA	↓ Lipid peroxidation
Iqbal et al. (13)	Mouse model Acute DOX (×1)	Telmisartan	NA	NA	↓	↓	NA	↓ Lipid peroxidation
								↓ Myocardial edema
Soga et al. (122)	Rat model Chronic DNR (3×/2 weeks)	Candesartan	↑	↔	↓	NA	↓	↓ 28 day mortality (50 vs. 19%)
								↑ %Factional shortening
								↑ E/A ratio
								↑ SERC2A transcription levels
Arozal et al. (123)	Rat model Chronic DNR	Olmesartan	↑	↓	NA	↓	NA	↓ Edema and hemorrhage on histopathology
								↓ AngII and AT-1R cardiomyocyte expression
								↑ %Fractional shortening
								↓ Metalloproteinase II expression
**BB**								
Chen et al. (124)	Mouse model Chronic DOX (every other day × weeks)	Carvedilol	↑	↓	↓	↓	↓	↑ Mitochondrial preservation
								↑ Cardiac stem cell expression
De Nigris et al. (125)	Rat model Chronic DOX/DNR (every other day × 12 days)	Nebivolol	↑	NA	NA	↓	NA	↑ Diastolic relaxation
		Carvedilol	↑	NA	NA	↓	NA	↑ Diastolic relaxation
**MRA**								
Lother et al. (24)	Mouse model Acute DOX (×1)	Eplerenone	↑	↓	↓	↔	↔	↑ Cardiac myocyte contraction and development gene expression (i.e., Ankrd1 and Nppa)
	Mouse model Chronic DOX (weekly ×5)	Eplerenone	↑	↓	↔	↔	↔	
Hullin et al. (25)	Mouse model Acute DOX (×1)	Eplerenone/MR gene ablation	↔	↔	↔	NA	NA	↑ Plasma aldosterone, ↑ AngII receptor, ↑ CTGF
	Mouse model Chronic DOX (weekly ×5)	Eplerenone/MR gene ablation	↔	↔	↔	NA	NA	↑ Plasma aldosterone, ↑ AII receptor, ↑CTGF
**ARNI**								
Boutagy et al. (29)	Rat model Chronic DOX (every 3 days × 3 weeks)	Sacubitril + valsartan	↑	↓	↓	NA	↔	↑ %Fractional shortening
								↓ Metalloproteinase activity
								↓ Myofibril vacuolization and inflammatory cell infiltration
**SGLT2i**								
Quagliariello et al. (33)	*In vitro* cell culture HL-1 mouse cardiomyocytes Acute DOX	Dapagliflozin	↔	↔	↔	↓	↓	↓ Pro-inflammatory cytokines IL-6, NF-kB and NLRP3
								↓ mTORC, FoxO1/O3a pathway expression
								↑ Cell viability
								↓ Ca^2–^ release
Sabatino et al. (34)	Mouse model Chronic DOX (Weekly ×5)	Empagliflozin	↑	↔	↓	NA	NA	↑ %Fractional shortening
								↑ Global longitudinal strain
								↓ Cardiac TnT and BNP levels
Quagliariello et al. (35)	Mouse model Chronic DOX (daily ×10) Mouse cardiomyocytes (HL-1)	Empagliflozin	↓	↔	↓	↓	↓	↓ IL-8, IL-6, IL-1β, NLRP3, and leukotriene B4
								↓ NF-kB activation
								↓ %Factional shortening
Barış et al. (36)	Rat model Chronic DOX (every other day × weeks)	Empagliflozin	↑	↓	↓	↔	↓	Normal QTc and PR intervals compared to prolonged in DOX toxicity
								↑ %Fractional Shortening
								↓ Myocardial edema, cell infiltration
**Dexrazoxane**								
Noel et al. (38)	Mouse model Chronic DOX (weekly ×6)	Dexrazoxane	↑	↔	↓	NA	NA	↑ Global longitudinal strain
Yu et al. (126)	Mouse model Chronic DOX (3× over 1 week)	Dexrazoxane	↑	NA	NA	NA	↓	↑ %Fractional Shortening
								↓ Activation of p38MAPK/NFkB apoptotic pathway
								↑ miR-15-5p mediated apoptosis
Jirkovsky et al. (127)	Rabbit model Chronic DNR (weekly ×10)	Dexrazoxane	↑	↓	↓	↓	NA	↑ Survival
								↓ Cardiac TnT levels
								↑ Mitochondrial preservation
								↑ Expression mitochondrial ANT1 and NRF1
**Statin**								
Riad et al. (48)	Mouse model Acute DOX ×1	Fluvastatin	↑	↓	NA	↓	↓	↓ TNFα expression
Sharma et al. (49)	Rat model Acute DOX ×1	Rosuvastatin	NA	NA	↓	NA	↓	↓ Na+ -K+ ATPase activity
								↓ DNA ladder formation
								↓ Cytoplasmic vacuolization
								↓ LDL and ↑ HDL
Huelsenbeck et al. (50)	Mouse model *in vivo* H9C2 rat cardiomyocytes *in vitro* Chronic DOX (weekly ×3)	Lovastatin	NA	NA	↓	↔	↓	↓ Cardiac TnT levels
								↓ DS DNA breaks and DNA damage
								↓ CTGF transcription
								↑ ANP levels
								↑ Doxorubicin antitumor activity in fibrosarcoma model
**Aerobic exercise**								
Alihemmati et al. (57)	Rat model Acute DOX ×1	High-intensity interval training	NA	NA	NA	NA	↓	↓ BAX/BCL2 levels
								↓ Caspase 6, GSK-3β levels
Wonders et al. (128)	Rat model Acute DOX ×1	Motorized treadmill	↑	↓	NA	↓	NA	
Ascensao et al. (129)	Rat model Acute DOX ×1	Motorized treadmill	NA	NA	NA	↓	↓	↑ HSP levels
								↓ Cardiac TnI levels
								↓ Cytoplasmic vacuolization
								↓ Mitochondrial swelling
Ascensao et al. (130)	Mouse model Acute DOX ×1	Swimming	NA	NA	NA	↓	NA	↓ Cardiac TnI levels
								↑ HSP60 levels

*ACE, angiotensin converting enzyme inhibitor; Akt, Protein kinase B; AngII, angiotensin II, Ankrd1, ankyrin repeat domain 1; ANT1, adenine nucleotide translocase type 1; ARB, angiotensin II receptor blocker; BAI-1, BAX activation inhibitor 1; BAX, Bcl-2-associated X protein; CTGF, connective tissue growth factor; DNR, Danorubicin; DOX, Doxirubicin; IL, interleukin; Fox, Forkhead box; GSK-3β, glycogen synthase kinase-3β; HSP, heat-shock protein; LV, left ventricle; MR, mineralocorticoid receptor; mTOR, mammalian target of rapamycin; mTORC, mammalian target of rapamycin complex; NA, not analyzed; NF-kB, Nuclear factor kappa B; NLRP3, NLR family pyrin domain containing 3; Nppa, Natriuretic Peptide A; NRF1, nuclear transcriptional factor 1; p38 MAPK, p38 mitogen-activated protein kinases; miR, miRNA encoding genes; PI3K, phosphoinositide 3-kinase; ROS, reactive oxygen species; TNFα, tumor necrosis factor alpha; TnI, troponin I; TnT, troponin T.*

**TABLE 2:** Summary of results from clinical studies for anthracycline induced cardiotoxicity therapies.

Study	Trial design	Follow up (mean/median)	Disease	Therapies	Findings (compared to placebo or control)				
					
					Δ LVEF	Myocardial strain	Ventricular remodeling (LVEDD, LVESD)	DD	Other
**ACE/ARB**									
Dessì et al. (17)	Phase II, placebo-controlled (*n* = 49)	18 months	BC, endometrial Ca, lymphoma, NSCLC, ovarian Ca	Telmisartan	↔	↑	NA	NA	↓ IL-6
									↓ ROS
Cardinale et al. (131)	Randomized, Placebo controlled (*n* = 114)	12 months	AML, lymphoma, MM, BC	Enalapril	↑	NA	↓	NA	↓ HF
									↓ Arrhythmia
Nakamae et al. (132)	Prospective, randomized controlled (*n* = 40)	0.25 months	Lymphoma	Valsartan	↔	NA	↓		↓ BNP
									↓ QTc
**BB**									
Kaya et al. (20)	Randomized, placebo-controlled trial (*n* = 45)	6 months	Breast cancer	Nebivolol	↑	NA	↓	NA	↓ BNP
CECCY Avila et al. (21)	Randomized controlled trial (*n* = 200)	6 months	Breast cancer	Carvedilol	↔	NA	↔	↓	↔ BNP
									↓Troponin I
Kalay et al. (133)	Randomized, placebo controlled (*n* = 50)	6 months	Lymphoma, BC	Carvedilol	↑	NA	↓	↓	
El-Shitany et al. (134)	Randomized, controlled trial (*n* = 50)	1 months	Pediatric ALL	Carvedilol	↑	↑	NA	↔	↓ Troponin I
									↓ LDH
**ACE/ARB ± BB**									
Georgakoppulos et al. (135)	Prospective, randomized controlled trial (*n* = 125)	12 months	Lymphoma	Enalapril	↔	NA	↔	↔	
				Metoprolol	↔	NA	↔	↔	
PRADA Gulati et	2 × 2 Factoria	Treatment	Breast cancer	Candesartan	↑	↔	NA	↔	↔ Troponin I and T
al. (15)	l, randomized, placebo-controlled trial (*n* = 120)	duration							↔ BNP
				Metoprolol	↔	↔	NA	↑	↑ BNP
				Combined	↔	↔	NA	↔	
PRADA Heck et al. (16)	2 × 2 factorial, randomized, placebo-controlled trial (*n* = 120)	23 months	Breast cancer	Candesartan	↔	↑	↓	NA	
				Metoprolol	↔	↔	↔	NA	
				Combined	↔	↔	↔	NA	
OVERCOME Bosch et al. (23)	Randomized, controlled trial (*n* = 90)	6 months	ALL, AML, lymphoma, MM	Carvedilol + Enalapril	↑	NA	NA	↔	↓ Death or HF
Liu et al. (136)	Randomized, controlled trial (*n* = 40)	4 months	Breast Cancer	Candesartan + Carvedilol	↑	NA	↓	NA	↑ ST and T wave abnormalities on ECG
									↓ Arrhythmia
									↓ Troponin
**MRA**									
ELEVATE Davis et al. (26)	Single center, randomized placebo-controlled trial (*n* = 44)	6 months	Breast cancer	Eplerenone	↔	NA	↔	↔	
Akpek et al. (27)	Randomized placebo-controlled study (*n* = 83)	3 week post-treatment	Breast cancer	Spironolactone	↑	NA	NA	↓	↓ Troponin I
									↓ TAC
**ARNI**									
Gregorietti et al. (31)	Prospective trial, serial patients on maximal GDMT (*n* = 28)	24 months	Breast cancer	Sacubitril/valsartan	↑	NA	↓	↓	↓ 6MWT
									↑ NYHA class
									↓ Mitral regurgitation
									↓ BNP
Martín-Garcia et al. (30)	Retrospective multicenter Spanish registry (HF-COH) (*n* = 67)	4.6 months	Breast cancer, lymphoma	Sacubitril/valsartan	↑	↔	↓	↔	↓ NYHA class
									↓BNP
**Dexrazoxane**									
Swain et	Multice	532 days	Breast	Dexrazoxane	↑	NA	NA	N	↓ Cardiac events
al. (39)	nter, double blinded RCT phase III (*n* = 682)		cancer					A	↓ Granulocyte and WBC count
	Multicenter, double blinded RCT phase III (*n* = 326)	397 days	Breast cancer	Dexrazoxane	↑	NA	NA	NA	↓ Cardiac events
									↓ Granulocyte and WBC count
Marty et al. (40)	Multicenter RCT phase III (*n* = 164)	126 days	Breast cancer	Dexrazoxane	↑	NA	NA	NA	↓ Cardiac events
									↓ Clinical HF
									↑ Cardiac-event free survival time
Asselin et al. (41)	Randomized placebo controlled study (*n* = 573)	9.2 years	Pediatric non-Hodgkin lymphoma, pediatric T-Cell ALL	Dexrazoxane	NA	NA	↓	NA	↓ Troponin T
									↑ %Fractional shortening
									↔ In infection, hematologic or CNS toxicity
Macedo et al. (44)	Meta-analyses of 7 RCTs and 2 retrospective studies from 1990 to 2019 (*n* = 2,177)	126 days to 7 years	Breast Cancer	Dexrazoxane	↑	NA	NA	NA	↓ Clinical HF
									↓ Cardiac events
									↔ Overall survival
Sun et al. (137)	Single center, single blinded RCT (*n* = 89)	126 days	Breast cancer w/concurrent T2DM	Dexrazoxane	↔	NA	↔	↓	↓ ROS levels
Ganatra et al. (47)	Single center, consecutive case series (*n* = 8)	13.5 months	T Cell lymphoma, NHL, AML, HL, Ovarian, Breast cancer with pre-existing asymptomatic LVEF <50%	Dexrazoxane	↑^#^	NA	NA	NA	↓ Symptomatic HF^#^
									↓ All cause mortality^#^
**Statin**									
Acar et al. (53)	Single center, randomized placebo-controlled trial (*n* = 40)	6 months	NHL, MM, leukemia	Atorvastatin	↑	NA	↓	NA	↓ Lipid levels
									↓ hsCRP levels
Seicean et al. (52)	Single center, retrospective study (*n* = 628)	2.6 years	Breast cancer	Uninterrupted statin therapy (any)	NA	NA	NA	NA	↓ Clinical HF incidence
Abdel-Qadir et al. (55)	Multicenter retrospective study (*n* = 1,332)	5.1 years	Breast cancer	Statin exposure (any) *	NA	NA	NA	NA	↓ LDL level
									↓ Hospitalization or ED visit for HF
Chotenimitkhun et al. (54)	Single center prospective cohort study (*n* = 51)	6 months	Breast cancer, leukemia, lymphoma	Prior statin therapy (any)	↑	↔	↓	NA	↔ Blood pressure
**Exercise**									
Kirkham et al. (64)	Randomized controlled trial (*n* = 24)	24–48 h	Breast cancer	Single session 30 min vigorous intensity exercise 24 h before ANT	↑	↑	↓	↔	↓ SVR, DBP, MAP, pulse
									↔ SV, CO
									↓ NT-proBNP
									↔ Troponin T
Kirkham et al. (65)	Randomized controlled trial (*n* = 24)	7–14 days	Breast Cancer	Single session 30 min vigorous intensity exercise 24 h before ANT	↔	↔	↔	↔	↔ Troponin T
									↔ NT-proBNP

*6MWT, 6 min walk test; ALL, acute lymphocytic leukemia; AML, acute myeloid leukemia; ANT, anthracycline; BC, breast cancer; BNP, brain natriuretic peptide; Ca, Cancer; CO, cardiac output; DBP, diastolic blood pressure; DD, diastolic dysfunction; DOX, doxorubicin; ECG, electrocardiogram; ED, emergency department; HF, heart failure; hsCRP, high sensitivity c-reactive protein; IL, interleukin; LDH, lactate dehydrogenase; LDL, low density lipoprotein; LVEDD, left ventricular end-diastolic diameter; LVESD, left ventricular end-systolic diameter; LVEF, left ventricular ejection fraction; MAP, mean arterial pressure; MM, multiple myeloma; NA, not analyzed; NHL, non-Hodgkin’s lymphoma; NT-proBNP, N terminal- pro hormone brain natriuretic peptide; NYHA, New York Heart Association; NSCLC, non-small cell lung cancer; ROS, reactive oxygen species; SV, stroke volume; SVR, systemic vascular resistance; TAC, total antioxidative capacity; T2DM, type 2 diabetes mellitus.*

*^#^Not statistically significant due to limited sample size.*

*2 or more statin prescriptions in 1 year prior to index date, 1 of the prescriptions containing index date.

### Angiotensin Converting Enzyme Inhibitors and Angiotensin Receptor Blockers

The renin-angiotensin-aldosterone system has been postulated to play an important role in the development of AIC. Doxorubicin has been shown to increase plasma levels of angiotensin II and increase local myocardial ACE activity, which has been linked to direct myocardial damage *via* myocyte apoptosis, fibrosis, inflammation, and development of ROS (11). Therefore, it is hypothesized that these therapies have a targeted effect in AIC beyond the typical role of ACE inhibitors (ACEI) and angiotensin receptor blockers (ARB) in neurohormonal regulation and ventricular remodeling in HF. Preclinical studies of ACEI and ARB demonstrated improved hemodynamics, improved cardiac remodeling, reduced incidence of heart failure, and decreased mortality in animal models (11–13). Collectively, these studies demonstrated that ACEI and ARB treatment decreased membrane lipid peroxidation, ROS production, and apoptosis in a variety of rat and mouse models (11). Studies have also compared the effectiveness of various ACEI/ARB therapies in AIC based on molecular structure and bioavailability. For example, zofenopril’s presence of a free-radical-scavenging sulfhydryl group and affinity for accumulation in cardiomyocytes provided more effective cardioprotection than enalapril and valsartan in rats (14).

Clinical trial data for ACE inhibitor and ARB therapy has been mixed. The PRADA (Prevention of Cardiac Dysfunction During Adjuvant Breast Cancer Therapy) trial was a 2 × 2 factorial, randomized placebo-controlled trial of adjuvant monotherapy and combined candesartan and metoprolol succinate administration during adjuvant epirubicin therapy in breast cancer patients. Early follow up results of this trial immediately following adjuvant therapy demonstrated that candesartan prevented a modest reduction in LV ejection fraction (LVEF) not seen with metoprolol or combination therapy (15). However, the two-year follow up of PRADA showed a modest decline in LVEF in all groups that was not attenuated by candesartan therapy compared to placebo. Compared to patients receiving candesartan monotherapy, patients in the placebo arm experienced a trend toward increase in LV end systolic volume and reduced global longitudinal strain (GLS) (candesartan, 0.2% [95% CI, -0.3 to 0.8] vs. no candesartan, 1.0% [95% CI, 0.5–1.5], *p* = 0.046 (16). A placebo-controlled randomized trial of telmisartan during epirubicin therapy similarly showed improved GLS at 18 months follow up in ARB-treated patients compared to placebo (17). Early levels of serum biomarkers of inflammation and oxidative stress, IL-6 and ROS, were increased compared to baseline in the placebo group but not the telmisartan group, indicating a potential mechanism of cardiotoxicity (17, 18). Additionally, there was an observed correlation between the decrease in GLS and the levels of IL-6 and ROS (17). Similarly, ARB therapy has also been shown to mitigate the production of ROS and inflammatory cytokines such as IL-6, with higher rates of LVEF recovery (17). This data indicates a potential protective role for ACE inhibitors and ARBs in patients at high risk for cardiotoxicity.

### Beta Blockers

Beta blockers play a key role in guideline directed medical therapy for treatment of HF due to their neurohormonal effects, reduction in heart rate, and attenuation of catecholamines and arrhythmias. However, some beta blockers, particularly carvedilol and nebivolol, have additional significant antioxidant effects that reduce ROS and prevent mitochondrial dysfunction, providing a specific advantage in prevention of AIC (19). An initial randomized trial of nebivolol showed higher LVEF, decreased LV end-systolic and end-diastolic diameters, and lower B-type natriuretic peptide (BNP) levels at 6 months than those receiving placebo (20). In the CECCY (Carvedilol Effect in Preventing Chemotherapy-Induced Cardiotoxicity) trial, the prophylactic use of carvedilol during anthracycline treatment had no impact on LVEF but an attenuation of increased troponin levels and protection against diastolic dysfunction compared to placebo (21). A meta-analysis of six RCTs evaluating prophylactic carvedilol monotherapy confirmed no effect on early changes in LVEF, but there was less increase in LV end-diastolic diameter, indicating a potential protective role in prevention of ventricular remodeling (22). In the beta blocker monotherapy arm of the PRADA trial, metoprolol succinate had no impact on decline of LVEF or GLS in short or long-term follow up (23). Importantly, unlike third generation beta blockers such as nebivolol and carvedilol, metoprolol has not been shown to have significant antioxidant properties. These studies indicate a potential role for beta blockers, particularly carvedilol and nebivolol, however, the small scale and short follow up of the majority of studies limits the wide applicability to all patients undergoing anthracycline therapy.

### Combination Therapy

The combination of beta blocker and ACE inhibitor therapy has been hypothesized to provide an increased synergistic effect compared to monotherapy, but the data is conflicting in different populations. In the PRADA trial which focused on breast cancer patients, combined candesartan and metoprolol did not exhibit significant change in LVEF, GLS, or LVESD compared to placebo both in short and long-term follow up (15, 16). Comparatively, the OVERCOME trial found a small benefit of combined enalapril and carvedilol in acute leukemia patients with no significant change in LVEF at 6 months, as compared to an average of 3% LVEF reduction in the placebo group (23). Further large trials are needed to evaluate the optimal patient population who may benefit from combined ACE inhibitor/ARB and beta blocker therapy.

### Mineralocorticoid Receptor Antagonists

Mineralocorticoid receptor antagonists (MRA) are another mainstay of heart failure treatment that has demonstrated conflicting preclinical and clinical results when applied to AIC. In a preclinical mouse model of AIC, both administration of eplerenone and direct cardiac myocyte mineralocorticoid receptor inhibition ameliorate the repressive effects of doxorubicin on cardiac myocyte transcription (24). However, in another mouse model of chronic AIC, eplerenone was not found to be cardioprotective particularly compared to enalapril (25). Clinically, the ELEVATE (Effect of Eplerenone on Left Ventricular Diastolic Function in Women Receiving Anthracyclines for Breast Cancer) trial was a randomized placebo-controlled trial of women with breast cancer to receive eplerenone during chemotherapy which showed no significant effect on LV systolic or diastolic dysfunction (26). Conversely, in Akpek et al., a placebo-controlled randomized controlled trial of spironolactone demonstrated that administration of spironolactone was protective of both systolic and diastolic cardiac function (27). There is an ongoing ATACAR (AuTophagy Activation for Cardiomyopathy Due to Anthracycline tReatment) study evaluating pravastatin and spironolactone in addition to maximally tolerated carvedilol and lisinopril for prevention of AIC (28). Further large-scale randomized controlled trials are needed to clarify the conflicting role of MRA in prevention of AIC.

### Angiotensin Receptor-Neprilysin Inhibitors

The angiotensin receptor-neprilysin inhibitor (ARNI), sacubitril-valsartan has been shown to have beneficial effects beyond ACE inhibitors and ARBs in the treatment of heart failure. In AIC, sacubitril-valsartan use in a rat model attenuated the decrease in ejection fraction with histological evidence of reduced myocardial toxicity and fibrosis in comparison with valsartan therapy alone (29). Although multiple small studies have evaluated the role of sacubitril-valsartan in treatment of symptomatic AIC, there is limited data regarding the role of sacubitril-valsartan in prevention of cardiotoxicity (30, 31). To this end, the PRADA II trial has been designed as a multicenter, randomized, placebo-controlled, double blinded clinical trial of sacubitril-valsartan therapy during epirubicin adjuvant chemotherapy to clarify the clinical cardioprotective role (32).

### Sodium-Glucose Transport Protein 2 Inhibitors

The newest studies of AIC in mice and *in vitro* human cells have demonstrated that empagliflozin and dapagliflozin protect against LV dysfunction with increased cardiomyocyte viability and reduction in cardiomyocyte apoptosis and inflammatory cytokine expression (33, 34). An *in vitro* cell based study by Quagliariello et al. demonstrated that administration of dapagliflozin reduced myocyte production of pro-inflammatory cytokines, including IL-1β, IL-8 (35). NF-kB and the NLRP3 inflammasome by 25–40%, as well as downregulation of the mTORC mediated pathway to apoptosis. In a rodent model, empagliflozin with doxorubicin administration led to preservation of LVEF and longitudinal strain with histologic evidence of reduced myocardial fibrosis and apoptosis (34–36). Given the mechanistic plausibility and promising preclinical studies of Sodium-Glucose Transport Protein 2 (SGLT2) inhibitors, clinical trials are needed to evaluate their potential role in prevention of cardiotoxicity.

### Dexrazoxane

In addition to the aforementioned conventional heart failure therapies, dexrazoxane was the first FDA approved therapy specific to AIC. Despite its early discovery, the mechanism of dexrazoxane cardioprotection continues to be elucidated. It was developed as a water-soluble analog of the iron chelator ethylenediaminetetraacetic acid (EDTA), aimed at reducing the production of ROS by anthracyclines *via* iron sequestration and iron displacement from anthracycline binding sites. However, more recent data involving dexrazoxane and its iron chelating metabolite ADR-925 suggested that topoisomerase IIβ inhibition and depletion, and not iron chelation, is responsible for its cardioprotective properties (37). Dexrazoxane also protects against cardiomyocyte apoptosis and necrosis *via* MAPK/NF-kB pathways inhibition, thereby reducing doxorubicin-induced inflammation (3). Serial cardiac MRI measurements in mouse models of anthracycline cardiotoxicity treated with dexrazoxane for 8 weeks demonstrated significantly less myocardial edema, improved GLS, and less myocardial deformation compared to no dexrazoxane (38).

Multiple randomized controlled trials have shown a reduced risk of cardiotoxicity with dexrazoxane administration during anthracycline therapy, primarily in children with hematologic malignancies and adults with breast cancer. Two double-blinded randomized controlled trials in advanced breast cancer patients demonstrated that dexrazoxane administered in a 10:1 ratio to anthracycline dose reduces the risk of LVEF decline and heart failure (39). Subsequent trials further solidified the role of dexrazoxane in the reduction in the risk of incident heart failure (11 vs. 1%, *p* < 0.05) and cardiac events (39 vs. 13%, *p* < 0.001) in advanced or metastatic breast cancer, without impeding tumor response rate (40). In children and adolescents with T cell acute lymphocytic leukemia (ALL) or non-Hodgkin lymphoma receiving anthracycline therapy, the addition of dexrazoxane improved LV wall thickness and fractional shortening on echocardiogram (41).

Unfortunately, these early studies indicated a possible increased risk of secondary malignancies and decreased antitumor efficacy (42). However, later studies confirmed the efficacy of dexrazoxane without the associated risk for secondary malignancies and antitumor efficacy (41, 43). The FDA approved usage of dexrazoxane, however, remains restricted to cardioprotection in advanced or metastatic breast cancer with ongoing anthracycline use after a cumulative dose of greater than 300 mg/m^2^ of doxorubicin. Off-label use of dexrazoxane in populations beyond metastatic breast cancer is increasing with promising results (44–46). Ongoing observational studies from the Children’s Oncology Group are evaluating the long term effects of dexrazoxane on heart failure and cardiomyopathy in children (NCT 01790152). Further studies are needed to further clarify the population of patients that may benefit most from prophylactic treatment with dexrazoxane.

Expanding on the role of dexrazoxane in primary cardiotoxicity prevention, a small case series followed eight patients with pre-existing asymptomatic LV systolic dysfunction for 13.5 months and found that two of three patients who were not treated with dexrazoxane developed cardiogenic shock, and all had markedly reduced ejection fraction (mean 42.5% baseline to 18% after chemotherapy (47). Patients treated with dexrazoxane had less significant LVEF reductions (mean baseline 39% to mean 34% post treatment) and none developed symptomatic HF. Future large scale clinical trials are needed to evaluate the cardioprotective effects of dexrazoxane in patients with established HF.

### Statins

In mouse models, fluvastatin and rosuvastatin were associated with improved cardiac function and reduction in cardiac inflammatory response, oxidative stress, and myocardial fibrosis (48, 49). Huelsenbeck et al. demonstrated that lovastatin co-treatment with anthracyclines reduces cardiotoxicity in mice *via* reduced mRNA levels and pro-fibrotic and pro-inflammatory cytokines (50). In comparison to carvedilol, rats pre-treated with rosuvastatin exhibited less LV fibrosis, LVEF reduction, and troponin elevation after 4 weeks of doxorubicin cessation (51). These pre-clinical studies demonstrate promise that the addition of statin therapy may aid in AIC prevention.

Several small single center studies have evaluated the use of statins for primary prevention of cardiotoxicity in breast cancer. In a single center observational cohort study of 628 women with breast cancer treated with anthracyclines, those on uninterrupted statin therapy throughout the 2.5 year follow up period demonstrated reduced incidence of HF (HR 0.3; 9% CI 0.1–0.9, *p* = 0.03) (52). In addition, in a randomized-controlled trial of 40 patients undergoing anthracycline therapy for various malignancies, prophylactic administration of statin for 6 months after anthracycline treatment was cardioprotective, with the placebo group experiencing significantly reduced LVEF (from 62.9 to 55.0%, *p* < 0.0001) while no change in LVEF was found in the atorvastatin group (from 61.3 to 62.6%, *p* = 0.144) on echocardiography (53). Similar results were obtained in a prospective study of 51 patients with various malignancies when assessed with CMR, where patients without statin therapy demonstrated a reduced LVEF (from 57.5 to 52.4%, *p* = 0.0003) while no significant change in LVEF was observed in patients receiving statin therapies (from 56.6 to 54.1%, *p* = 0.15) after 6 months (54). In a propensity score-matched cohort study, breast cancer patients on anthracycline therapy who had received statin therapy had significantly lower rates of heart failure hospital admissions (HR 0.45 95% CI 0.23–0.85, *p* = 0.01) (55). Additional, robust randomized controlled trials evaluating the efficacy of statins in AIC, including the Preventing Anthracycline Cardiovascular Toxicity with Statins (PREVENT; NCT01988571) and the Statins TO Prevent the Cardiotoxicity from Anthracyclines (STOP-CA; NCT02943590) trials are ongoing.

### Aerobic Exercise

Recent literature suggests that aerobic exercise may be particularly beneficial in mitigating the cardiotoxic effects of anthracyclines due to the stimulation of antioxidants, reduction of ROS levels, and decrease in activation of apoptotic pathways. Aerobic exercise leads to cardioprotective remodeling by promoting myofilament synthesis and physiologic hypertrophy in various animal models (56). In addition, Alihemmati et al. demonstrated that high intensity interval training in rats treated with doxorubicin led to a reduction in pro-apoptotic proteins, such as BAX and caspase 6, and overall fewer apoptotic cells compared to controls (57). Other pre-clinical studies have shown the benefit of exercise in reducing ROS production, increased gene expression of antioxidant molecules, reduced mPTP susceptibility, increased mitochondrial resilience, and lowered lipid peroxidation in rat models of AIC (58). A variety of pre-clinical studies demonstrated that swimming, forced treadmill and wheel running exercise programs reduced fibrosis and necrosis, decreased myocardial inflammatory markers, and protected against loss of cardiomyocytes and myofibrils among rodents exposed to doxorubicin (59).

In humans, evidence of aerobic exercise in protecting against AIC is limited. Studies have consistently demonstrated that anthracycline therapy reduces cardiorespiratory fitness in adult breast cancer patients and pediatric patients with hematologic malignancies (60, 61). Additionally, exercise programs are feasible and can improve patient reported cardiorespiratory tolerance and peak VO_2_ (62, 63). Despite this, high-quality data demonstrating that aerobic exercise protects against alterations in objective parameters of cardiotoxicity are lacking. Kirkham et al. demonstrated that a single 30-min session of vigorous intensity exercise prior to doxorubicin exposure was associated with improved LV volumes, improved GLS and lowered circulating pro-BNP values compared to controls within 48 h of anthracycline administration (64). However, these protective effects were no longer seen at 2 weeks after anthracycline administration (65). These findings are limited by a short follow up period, small sample size, and confounded by the effects of exercise itself on pro-BNP levels and myocardial strain. Furthermore, these results do not evaluate the effect of longer-term exercise programs on AIC.

Several multicenter studies are currently evaluating the cardioprotective effects of exercise in AIC. The ONCORE study (Exercise-based Cardiac Rehabilitation for the Prevention of Breast Cancer Chemotherapy-induced Cardiotoxicity; NCT03964142) (66), the ATOPE Trial (Attenuating cancer Treatment-related Toxicity in Oncology Patients with a Tailored Physical Exercise Program; NCT 03787966) (67), and the BREXIT trial (Breast Cancer Exercise InTervention) (68) are randomized controlled trials aimed at determining whether exercise-based cardiac rehabilitation in breast cancer patients can mitigate anthracycline cardiotoxicity measured by LVEF and GLS, cardiac biomarkers, and peak VO_2_. The results of these robust studies may eventually help define the degree by which aerobic exercise reduces risk for anthracycline induced cardiotoxicity. If the studies demonstrate positive results, they may justify cardiac rehabilitation before or after anthracycline therapy as standard of care even in the absence of overt cardiac dysfunction.

## Treatment of Anthracycline Induced Cardiomyopathy

### Alterations in Chemotherapy Regimen

**FIGURE 3:**
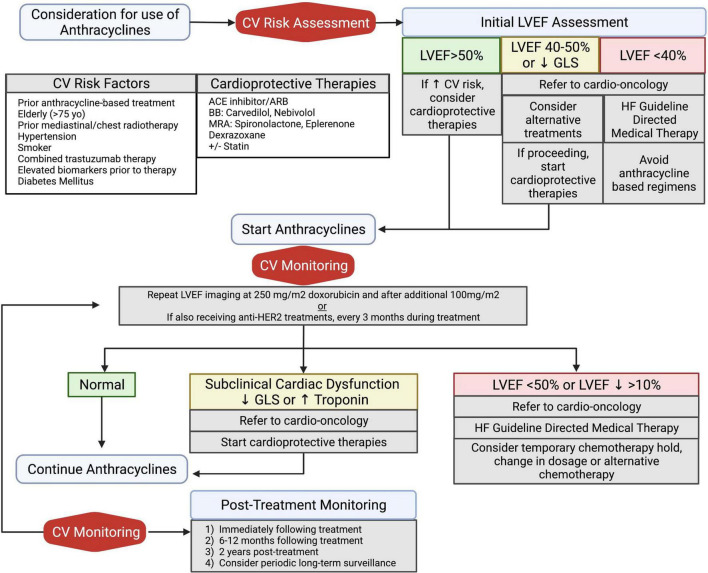
Algorithm of clinical management for prevention and treatment of anthracycline induced cardiotoxicity. All patients should undergo baseline cardiovascular risk assessment, including an echocardiogram. Initiation of cardioprotective medications should be considered in patients with increased cardiovascular risk or abnormal baseline LVEF assessment. Patients with high-risk anthracycline therapy due to high dose (250 mg/m^2^ doxorubicin) and concomitant anti-HER2 treatments should undergo serial cardiovascular monitoring during treatment. All patients should undergo post-treatment LVEF monitoring for detection of long-term cardiovascular sequelae. Adapted from ESMO 2020 guidelines (75). ACE, angiotensin converting enzyme; ARB, angiotensin receptor blocker; BB, beta blocker; CV, cardiovascular; GLS, global longitudinal strain; HF, heart failure; LVEF, left ventricular ejection fraction; MRA, mineralocorticoid receptor antagonist. Created with BioRender.com.

As patients undergo treatment with anthracyclines, cardio- vascular monitoring is warranted both during the treatment period and as part of post-treatment surveillance (Figure 3). If early signs of cardiotoxicity develop, such as reductions in LVEF or decreased GLS, chemotherapy regimen changes must first be considered as an interdisciplinary team. While prevention or reversal of cardiotoxicity would ideally involve withholding or delaying anthracycline administration, this is often not practical given the risk of malignancy progression.

Additional considerations include changing the anthracycline agent and altering the anthracycline infusion schedule. Early results by Hortobagyi et al. demonstrated that continuous infusion strategies were associated with more than a 75% decrease in the frequency of congestive heart failure in cumulative doses greater than 450 mg/m^2^ (69). However, later studies demonstrated equivocal results. In a study comparing continuous intravenous vs. bolus infusion of doxorubicin among breast cancer and sarcoma patients receiving doxorubicin, bolus infusion was associated with lower rates of cardiac events 1–5 years after treatment (4 vs. 5.1% at 1–5 years after treatment, *p* = 0.046), but no significant change was noted for cardiac events within 1 year of treatment or more than 5 years after treatment (6.5 vs. 5.6%, *p* = 0.098 and 0.5 and 0.9%, *p* = 0.068) (70). Similarly, bolus and continuous infusion of doxorubicin among pediatric patients with acute lymphoblastic leukemia demonstrated no difference in rates of cardiotoxicity measured on serial echocardiography monitoring for 8 years (71). Alterations in the anthracycline agent itself may also be considered. Pegylated liposomal doxorubicin has been developed to reduce myocardial anthracycline concentrations, and has been shown in a meta-analysis to be associated with a significant reduction in cardiotoxicity (OR 0.46 95% CI 0.23–0.92) (72). Different anthracycline agents have variable profiles of cardiotoxicity, such as danorubicin and epirubicin showing less long-term cardiotoxicity than doxorubicin in childhood cancer survivor cohorts (72). Thus, agent switching can be employed where applicable to minimize the effects of AIC.

### Neurohormonal Pharmacologic Therapy

In patients undergoing anthracycline therapy, serial echocardiography and clinical monitoring for development of LV dysfunction and heart failure is indicated to ensure timely treatment and consideration of changes in chemotherapy regimens. In AIC, the LVEF threshold for initiation of pharmacologic therapy is shifted from the conventional cutoff of an LVEF of 40% to LVEF of less than 50% due to the increased risk of further decline in LVEF in this population. There is evidence for the effectiveness of traditional HF guideline directed medical treatment (GDMT) in this population. In patients with new LVEF of <45%, initiation of enalapril and carvedilol led to complete recovery in 42%, partial recover in 13% and no change in 45% of patients (73). A prospective study of patients undergoing anthracycline chemotherapy monitoring for decline of LVEF showed that for AIC defined as LVEF of <50%, early diagnosis and initiation of combined enalapril and beta blocker therapy has been associated with greater LVEF recovery (74).

Additionally, the role of ARNIs is being elucidated. In a small study of 28 patients who developed AIC and were previously optimized on GDMT, patients were transitioned to sacubitril-valsartan with significant improvement in LVEF, ventricular remodeling and diastolic function indicating a promising role for sacubitril-valsartan (31). A retrospective multicenter registry study identified sixty-seven patients started on sacubitril/valsartan for AIC and similarly found an improvement in LVEF, ventricular remodeling and NT-proBNP levels after initiation of therapy (30).

Current treatment of AIC is largely based on observational studies and adaptation of existing HF guidelines are without specific randomized trials comparing the relative advantages of different guideline-directed therapies in this unique patient population, particularly newer agents such as ARNIs and SGLT2i. Current available data have been adapted into clinical surveillance and management guidelines and consensus statements released from the European Society for Medical Oncology (ESMO) and the American Society of Echocardiography (ASE), respectively (75, 76). These guidelines have been summarized into a clinical algorithm proposed in Figure 3.

### Tolerability Considerations of Pharmacologic Therapy

Compared to conventional heart failure patients, oncology patients with AIC represent a unique cohort of patients vulnerable to hemodynamic intolerance of neurohormonal therapies due to the anemia, dehydration, fatigue and hypotension often associated with their underlying malignancy and treatment regimen. Unfortunately, the tolerability of guideline directed medical therapy in the AIC population is not well-studied. The CECCY trial reported that symptomatic hypotension was the most common side effect of carvedilol administration, affecting three patients (3.1%). While this was an overall low incidence of hypotension, approximately only 9% of the carvedilol arm achieved the dosing goal of 50 mg/day (77). The PRADA trial had reported that candesartan and metoprolol were generally well-tolerated amongst trial participants, but had excluded patients with underlying hypotension with SBP < 110 as well as any history of beta blocker and ACE/ARB intolerance (15, 16). As the PRADA II trial evaluates the efficacy of ARNI in the treatment of AIC, it will be important to understand the potential potent hemodynamic effects of these agents on populations most vulnerable to hemodynamic fluctuation. Future studies must carefully select populations representative of cancer patients on AIC and compare hemodynamic tolerability of neurohormonal therapy in this population with those of conventional heart failure. Additionally, it remains unclear if optimal or target dosing of medications differs in these patients compared to the general HF population.

### Cardiac Resynchronization Therapy

In patients with AIC and standard guideline indications for cardiac resynchronization therapy (CRT), CRT has been found to be safe and effective. CRT reduces myocardial tissue desynchrony, which leads to alterations in perfusion and metabolism that may be particularly beneficial for myocyte toxicity in patients with AIC (78). Small studies, including Multicenter Automatic Defibrillator Implantation Trial-Chemotherapy Induced Cardiomyopathy (MADIT-CHIC), have demonstrated that CRT in AIC patients with dilated cardiomyopathy and left bundle branch block was associated with improvement in LVEF and New York Heart Association (NYHA) functional class during the follow up period (79–81). In retrospective case control studies, patients with AIC and CRT showed evidence of ventricular remodeling (80) and long term mortality (82) similar to patients with alternative etiologies of non-ischemic cardiomyopathy.

### Mechanical Circulatory Support

Treatment of end-stage cardiomyopathy may include consideration for advanced heart failure therapies, including ventricular assist devices (VAD) and orthotopic heart transplant (OHT). Evaluation for these invasive therapies can be complicated due to the patient’s oncologic history including ongoing chemotherapies, prognosis or, if in remission, risk of recurrence. The use of VADs is still relatively rare and AIC was the etiology of cardiomyopathy in only 0.5% of registry patients on mechanical circulatory support (MCS) (83–85). Compared to idiopathic dilated cardiomyopathy and ischemic cardiomyopathy, AIC patients undergoing left-sided VAD (LVAD) implantation were more likely to be female and more likely to have LVAD as destination therapy (86). One major limiting factor for LVAD implantation is the high risk of right ventricular dysfunction in AIC, which increases the risk for right ventricular failure after LVAD implantation. In fact, in an Interagency Registry for Mechanically Assisted Circulatory Support (INTERMACS) analysis, almost one fifth of patients required biventricular MCS (87). Despite the increased need for biventricular support, survival is similar between AIC patient and other causes of cardiomyopathy and indicates that LVAD may yield acceptable outcomes in those with active cancer (86, 87). Hence, it may be a viable strategy not only in conventional use as support in those with end-stage HF from AIC, but perhaps in highly selected individuals as a bridge during cancer treatment (88).

### Orthotopic Heart Transplant

Although OHT remains the definitive treatment for end-stage heart failure, patients with AIC often face barriers to candidacy due to their history of malignancy and concern for recurrence on immunosuppressive medications. To date, 0.8–2.5% of all OHT patients had chemotherapy associated cardiomyopathy (89). OHT recipients with AIC were younger, female, and less likely to have bridge-to-transplant LVAD. Most notably, survival rates after OHT in patient with AIC have been found to be comparable to dilated and ischemic cardiomyopathy (90, 91). Additional studies are needed to better understand how prior malignancy may impact OHT survival and to investigate the role of alternative immunosuppressive regimens that may be useful in this population.

## Investigational Therapies

In addition to the investigation into applications of the aforementioned cardioprotective therapies to the treatment of AIC, efforts have been made to design therapies targeting the specific cardiotoxic mechanisms of anthracyclines. However, molecular target selection has proven challenging due to the heterogeneous effects of anthracyclines on many cellular constituents, and the need to select pathways that do not compromise chemotherapeutic efficacy, or cause significant off-target effects. Despite these challenges, several investigational therapies have been proposed, and several with promising preliminary results that are summarized in Table 3.

**TABLE 3:** Summary of findings from clinical and preclinical studies for investigational therapies.

Study	Study design	Anthracycline	Therapies	Findings (compared to anthracycline + no therapy)
**Targeting apoptosis**				
Amgalan et al. (93)	Mouse model Zebrafish model	Acute DOX (×1) Chronic DOX (every other day × weeks)	BAI-1	↓BAX translocation
				↓ Cardiomyocyte necrosis and apoptosis
				↑ Cardiac contraction
				↓ Pericardial edema
Fisher et al. (94)	Mouse model	Acute DOX (×1)	Sildenafil	↓ *In vivo and in vitro* apoptosis measured by TUNEL
				↑ Bcl-2 expression
				↓ Myofibrillar disarray on immunofluorescence
				↓ ST interval prolongation
				↓ Mitochondrial membrane potential dissipation
				↑ Myocardial contractility
Liu et al. (95)	Mouse model	Acute DOX	Melatonin	↑ Antioxidant activity measured by fluorescence assay
				↑ 5 day survival
				↑ *In vitro* cardiomyocyte function by HR and LVDP
				↑ *In vivo* cardiac function by LVEDP, LVESP, and dP/dt measurements
				↓ Histological cytoplasmic vacuolization, mitochondrial damage, and swelling
				↓ Apoptosis by TUNEL
Yang et al. (97)	*In vitro:* H9c2 rat cardiomyoblasts *In vivo:* mouse model	Chronic DOX (Every other day × weeks)	Melatonin	↓ AMPkα2 activation
				↓ ROS generation
				↑ ATP production
				↓ Apoptosis
				↑ Mitochondria length
				↓ mitochondrial fragmentation
**Targeting oxidative stress and inflammation**				
Siveski-Iliskovic et al. (98)	Rat model	Chronic DOX (6×/2 weeks)	Probucol	↓ LVEDP, ↑LVEF ↑LVSP
				↑ Levels of antioxidant enzymes GPx and SOD
				↓ Lipid peroxidation
				↓ Mitochondrial swelling, cytoplasmic vacuolization, lysosomal body, sarcotubular deformation
Cao and Li. (99)	*In Vitro:* H9c2 rat cardiomyocytes	Acute DOX	Resveratrol	↑ Antioxidant enzymes SOD, catalase, GSH, and GR activity
		Xanthine		↓ Cardiomyocyte ROS
		HNE		↑ Cell viability
Tatlidede et al. (100)	Rat model	Chronic DOX (every other day × weeks)	Resveratrol	Echo: ↑ LVEF; ↑ %FS ↓LVEDD, ↓LVESD ↑ relative wall thickness
				↓ LDH ↑ CK ↑ AST
				↑ Antioxidant enzyme levels of catalase, SOD, GSH
				↓ ROS
				↓ Capillary vasocongestion and cytoplasmic vacuolization
Liu et al. (101)	*In vitro:* H9c2 rat cardiomyocytes	Acute DOX x1	Resveratrol	↓ Cell apoptosis
				↑ Cell viability
				↓ Pro-apoptotic protein expression of FoxO1, p53, and Bim
				↓ ROS production
				↑ SOD activity
Monahan et al. (102)	*In vitro:* H9c2 rat cardiomyocytes	Acute DOX x1	Resveratrol	↓ ROS production
				↑ Cell viability (compared to no therapy and compared against carvedilol and dexrazoxane)
Chen et al. (103)	Rat model	Chronic DOX (every 3 days × 3 weeks)	Co-enzyme Q10	↓ Fibrosis on tissue trichrome staining
				↓ Pro-fibrotic CTGF and TGF-β1, MMP2, MMP9, COL1A1 levels
				↓ Pro-apoptotic Bak, BAX, caspase-9, caspase-3 levels
				↓ Apoptosis measured by TUNEL
Akolkar et al. (104)	Rat model	Chronic DOX (6×/3 weeks)	Vitamin C	Echo: ↑ LVEF ↑ %FS ↓ E/A ratio
				Histology: ↓ cytoplasmic vacuole formation; ↑myofibrils ↓ fibrosis s
				↓ Lipid peroxidation; ↓ ROS
				↑ Antioxidant enzyme expression of SOD, GPx and catalase
				↓ TNFα, IL-1β, IL-6
				↓ Proapoptotic Bnip-3, Bak, BAX and caspase-3
				↓ Inflammatory response associated JNK, NF-kB, and IKK levels
				↑Akt and STAT3 levels
Berthiaume et al. (105)	Rat model	Chronic DOX (weekly ×7)	Vitamin E	↔ Mitochondrial oxygen consumption
				↔ Ca loading capacity
				↔ Cardiomyocyte damage and cytoplasmic vacuolization
				↓ ROS protein carbonyls
Arica et al. (106)	Rat model	Acute DOX ×1	N-acetylcysteine	↓ MDA
				↓ AST, LDH, CK
				↔ SOD
				↓ Cytoplasmic vacuolization ↓ myofibril disarray ↓ myofibril loss
Unverferth et al. (107)	Dog model	Chronic DOX (weekly ×16)	N-acetylcysteine	↔ LVEF
				↔ CI, LVEDP, MAP
				↓ Subendocardial and subepicardial fibrosis
EPOCH trial Jo et al. (108)	Single center, randomized controlled clinical study (*n* = 103) 12 months follow up	DOX Epirubicin	N-acetylcysteine	↔ Troponin I ↔ CK-MB
				↔ LVEF decline ↔LVESD ↔LVEDD, ↔E/A ratio, ↔ E/E’
	**Population:** adults w/BC, lymphoma			↔ All cause mortality
**Inhibiting CYP1**				
Asnani et al. (109)	*In vivo:* Mouse model, zebrafish model	Acute DOX	CYP1 inhibition/Visnagin	↓ Induction of CYP1 enzymes
	*In vitro:* HL-1 mouse cardiomyocytes			↓ Cardiomyocyte apoptosis
Lam et al. (110)	Zebrafish model	Acute DOX	CYP1 inhibition w/various genes	↓ Pericardial edema
				↑ Cardiac contraction
				↑ Blood flow
**Use of Stem Cells**				
Bolli et al. (112)	Open-label, phase I, double blind, placebo, randomized control trial (*n* = 31)	DOX	Allo-MSC	↔ Cardiovascular death ↔ HF hospitalization
	**Population:** adults w/leukemia, BC, HL, NHL, sarcoma with chronic AIC	Epirubicin, Danorubicin		↔ LVEF, ↔ GLS, ↔LVEDV, ↔ LVESV
				↔ Scar tissue
				↔ NT-proBNP
				↓ MLHFQ score
				↓ 6MWT*
O’Brien et al. (113)	SENECA trial patient specific iCMs	DOX	Extracellular vesicles from MSCs	↑ Cardiomyocyte viability
				↑ ATP production
				↓ ROS production
				↑ Pro-mitochondrial biogenesis associated PGC-1α

*6MWT, 6 minute walk test; Akt, Protein kinase B; AIC, anthracycline induced cardiotoxicity; AMPkα2, AMP-activated protein kinase catalytic subunit alpha-2; AST, aspartate transaminase; ATP, adenosine triphosphate; BAI-1, BAX activation inhibitor 1; Bak, Bcl-2 homologous antagonist/killer; BAX, Bcl-2-associated X protein; Bc, breast cancer; Bcl-2, B-cell lymphoma 2; BIM, Bcl-2-like protein 11; Bnip-3, Bcl2/adenovirus E1B 19 kDa protein-interacting protein 3; CI, cardiac index; CK, creatine kinase; COL1A1, collagen type 1 alpha 1; CTGF, connective tissue growth factor; CYP1, cytochrome P450 family 1; DOX, doxorubicin; FS, fractional shortening; Fox, forkhead box; GSH, glutathione; GPx, glutathione peroxidase; GR, glucocorticoid receptor; HR, heart rate; HL, Hodgkin’s lymphoma; HNE, 4-hydroxy 2-non-enal; iCM, induced cardiomyocyte; IKK, IkB kinase; IL, interleukin; LVDP, left ventricular diastolic pressure; JNK, C-Jun N-terminal kinease; LVEDP, left ventricular end diastolic pressure; LVESP, left ventricular end systolic pressure; LVSP, left ventricular systolic pressure; LVEDV, left ventricular end diastolic volume; LVESV, left ventricular end systolic volume; MAP, mean arterial pressure; MDA, malondialdehyde; MLHFQ, Minnesota living with heart failure questionnaire; MMP, metalloproteinase; MSC, mesenchymal stem cells; NFkB, nuclear factor kappa B; NHL, non-Hodgkin’s lymphoma; NT-proBNP, N terminal- pro hormone brain natriuretic peptide; PGC-1α, proliferator-activated receptor gamma coactivator 1-alpha; ROS, reactive oxygen species; SOD, superoxide dismutase; STAT3, signal transducer and activator of transcription 3; TGF, transforming growth factor; TNF, tumor necrosis factor; TUNEL, Terminal deoxynucleotidyl transferase dUTP nick end labeling.*

*Trend toward statistical significance.

### Therapies Targeting Apoptosis

Ongoing investigations are aimed at attenuating the release of apoptotic factors that are integral to the pathophysiology of AIC. Bcl-associated X protein (BAX) is a member of the BCL-2 protein family that has been shown to promote both cellular apoptosis and necrosis. With cellular stress exposure, BAX translocates to the mitochondrial membrane and increases outer membrane permeability to release pro-apoptotic factors and promotes inner membrane mPTP opening to promote cellular necrosis (92, 93). Amgalan et al. utilized BAI1, a small molecular inhibitor of BAX, and demonstrated that the prevention of BAX translocation to the outer mitochondrial membrane leads to reduced apoptotic and necrotic cardiomyocyte death in murine and zebrafish animal models (94). On a cellular level, treatment with BAI1 helped to maintain mitochondrial integrity and ultimately protected against LV systolic dysfunction as measured by echocardiographic parameters (94). Interestingly, BAX inhibition *via* BAI1 did not adversely affect anthracycline cancer cell toxicity in mice; presumably from markedly higher levels of BAX in tumor cells compared to cardiomyocytes.

The use of the phosphodiesterase-5-inhibitor, sildenafil, has also demonstrated anti-apoptotic effects. Pre-treatment with sildenafil prior to doxorubicin initiation in mice was associated with preserved LV function and decreased cellular apoptosis (95). *In vitro* analysis of the underlying mechanisms revealed that sildenafil preserved mitochondrial membrane potentials, reduced intracellular levels of the pro-apoptotic caspase-3 enzyme, and preserved levels of anti-apoptotic Bcl-2 family proteins. These results indicate that sildenafil may be useful in the modulation of apoptotic pathways associated with AIC, however, the safety of the use sildenafil without compromising antitumor efficacy must be better delineated.

Another apoptotic pathway observed in AIC is *via* AMP-activated protein kinase α2, or AMPKα2. AMPKα2 is overexpressed in fibroblasts treated with doxorubicin and has been shown to activate the transcription of pro-apoptotic molecules including p27, Apaf-1, and Bim. Several studies have shown that melatonin is cardioprotective against AIC, owing to its role as a potent antioxidant and free radial scavenger (96, 97). These studies also suggested that melatonin may also protect against apoptotic cardiomyocyte death, and recent data show that one of the potential mechanisms of this may be *via* inhibition of the upstream pathways of AMPKα2 transcription (97).

### Therapies Targeting Oxidative Stress and Inflammation

Multiple antioxidant therapies have been investigated to relieve the oxidative stress and ROS production that is a hallmark in the pathogenesis of AIC. In addition to the antioxidant effects of melatonin as mentioned above, the lipid-lowering agent probucol has been associated with strong antioxidant properties. Probucol administration was shown to increase ROS degradation, which led to prevention of cardiac remodeling, normalization of LV systolic function, and improved overall survival in a murine model of AIC (98). Resveratrol has also demonstrated antioxidant effects that translate to reduced fibrosis and inflammation in cardiomyocytes (99). Resveratrol has been shown to reduce lipid peroxidation and increase mitochondrial stability as prophylactic treatment in rat and mouse models of AIC (100, 101). In fact, Monahan et al. demonstrated that prophylactic resveratrol was associated with increased cell survival and decreased ROS production compared to dexrazoxane and carvedilol in *in vitro* rat cardiomyoblast models (102). Co-enzyme Q10 has also been studied in an *in vivo* rat model of AIC, Q10 co-administration increased cellular survival and reduced fibrosis in cardiomyocytes (103).

In addition, vitamins with potent antioxidant properties have been studied in preclinical AIC models. Prophylactic and co-administration of vitamin C with doxorubicin was shown to increase the LV fractional shortening and LVEF on echocardiography, reduce fibrosis and myofibril loss, and improve overall rat survival *via* reduced lipid peroxidation and superoxide anion production (104). However, while administration of vitamin E in rat models of AIC was also associated with decreased ROS production, it failed to prevent mitochondrial dysfunction or cardiac remodeling on histopathology (105).

While the results above demonstrate generally promising results, studies involving N-acetylcysteine (NAC), a free radial scavenger, have been mixed. While rat models of AIC treated with NAC demonstrated improved myocardial architecture and reduced levels of cardiotoxic biomarkers (106), these improvements were not observed in canine models (107). In addition, NAC was not associated with any improvement in AIC as measured by echocardiographic measures and cardiac biomarkers in a prospective randomized controlled trial in breast and lymphoma patients (108). While the studies on the aforementioned antioxidants suggest that targeting oxidative stress may be cardioprotective in AIC, the equivocal NAC data suggest it is first necessary to determine the most effective antioxidant agents and translate these findings to meaningful clinical parameters.

### Cytochrome P450 Family 1 Inhibition

In a zebrafish model of AIC, Asnani et al. demonstrated that doxorubicin treatment significantly increased levels of cytochrome P450 family 1 (CYP1) enzymes, and that various substances inhibiting the CYP1 pathway attenuated cellular apoptosis and exhibited less features of cardiomyopathy under electron microscopy (109). Lam et al. extended these findings to demonstrate that both molecular inhibition of CYP1 and gene deletion of CYP1A prevented declines in myocardial contractility and perfusion compared with controls in zebrafish treated with doxorubicin (110). Further study is needed to clarify the role of CYP1 inhibition in doxorubicin related anthracycline toxicity, such as whether it is involved in the metabolism of doxorubicin itself or in the clearance of cardiotoxic metabolites.

### Use of Stem Cell Therapies

The Stem Cell Injection in Cancer Survivors (SENECA) trial is an ongoing first in-human randomized controlled trial assessing the effects of allogenic bone marrow derived mesenchymal stromal cell (MSC) administration in the treatment of AIC in breast cancer patients (111). The primary endpoints are assessment of cardiac function on cardiac MRI, cardiac biomarkers, and quality of life questionnaires. To date, the trial has demonstrated no significant difference between the treatment and control groups with regard to clinical outcome, but with a trend toward significantly improved in 6-min walk time (6MWT) (*p* = 0.056) and quality of life questionnaires (*p* = 0.048) in the MSC group (112). In addition, O’Brien et al. demonstrated that in patient-derived cardiomyocytes (iCM) that underwent injury with anthracyclines, treatment with mitochondria-rich extracellular vesicles from mesenchymal stem cells improved mitochondrial biogenesis and contractility, with lower production of ROS (113). While preliminary, these data suggest that mesenchymal stem cell targeting of mitochondrial transfer may be a viable target for reducing anthracycline induced cardiotoxicity.

### Use of Gene Therapy

While anthracycline cardiac toxicity is generally dose-dependent, some patients are able to tolerate high doses of anthracyclines, while others develop AIC at relatively lower doses. Differences in patient susceptibility invite consideration of genetic variables that either predispose to or protect against AIC. Several genetic variants have been identified through genomic analysis of both animal models and human trial participants (114–116). These genes affect the synthesis of various important proteins in the mechanisms of AIC, such as the variant *HAS3* that produces the reactive oxygen species neutralizer hyaluronan and *RARG* that derepresses topoisomerase 2β (116).

Gene therapy techniques are rapidly being applied to various disease pathologies, including heart failure, and may have important implications on the future of AIC treatment. The first clinical trial of cardiac gene therapy in conventional heart failure, CUPID (Calcium Up-Regulation by Percutaneous Administration of Gene Therapy in Cardiac Disease) utilized an adeno-associated virus (AAV) vector to carry sarcoendoplasmic reticulum calcium ATPase (SERCA2a) transgenes in an attempt to increase cardiac contractility (117). While the phase 2B endpoints were non-significant, CUPID encouragingly demonstrated the safety and feasibility of gene therapy in a large cohort of 250 heart failure patients (118). Kok et al. applies the concepts of cardiac gene therapy to the theoretical treatment of AIC, and proposes methods of developing cardiotropic gene vectors and *in vivo* preclinical delivery models to 1 day feasibly deliver cardioprotective genes to cardiomyocytes while minimizing effects on off-target cancer cells (119). While the concept of gene therapy in AIC is still in its infancy, with further research into appropriate cardioprotective genetic variants and effective gene delivery, gene therapy may one day revolutionize the treatment of AIC.

## Conclusion and Future Directions

The current treatment of AIC is largely extrapolated from conventional neurohormonal pharmacologic therapy established as guideline directed medical therapy for heart failure with reduced ejection fraction, and evidence of the efficacy of these therapies specific to AIC are primarily from small clinical trials with limited follow up duration. Moreover, a meta-analysis of these clinical trials for neurohormonal therapy in AIC demonstrate only a modest improvement in LV function, and interpretation was substantially limited by highly heterogeneous populations that were studied (120). Thus, large-scale, randomized clinical trials are needed to firmly establish the benefit of these therapies in AIC by targeting hard clinical endpoints and focusing on higher risk populations, including focus on patients with cardiovascular risk factors and/or hematologic malignancies.

The lack of clinically significant improvement with neurohormonal therapy in AIC also raises consideration for the impact of unaddressed pathophysiologic differences between AIC and conventional heart failure. With the exception of dexrazoxane, clinically applicable therapeutics specific to anthracycline cardiotoxicity are still lacking. Though various preclinical studies have demonstrated promising potential therapeutic targets specific to the mechanisms of AIC, further clinical and preclinical studies are needed to substantiate their potential benefit. In addition, the current fragmented understanding of AIC pathophysiology impairs the development of more effective therapies. As the mechanisms of AIC continue to be elucidated, attempts to unify the connections between the various cardiotoxic mechanisms in AIC will help identify potential therapeutic targets that simultaneously address various pathways of toxicity. Further consideration of differences in the genetics and cardiovascular risk profiles of patients with AIC compared to conventional heart failure will help unveil individualized treatment options.

## Author Contributions

JV, AS-M, RC, and EY contributed to the conception of review article content, review and editing of the manuscript. JV and AS-M wrote the first draft of the manuscript and created figures and tables. All authors have read and approved the final version of the manuscript.

## Conflict of Interest

EY reports research funding from CSL Behring, Boehringer Ingelheim, and Eli and Lilly for research outside the current manuscript, and reports consulting fees from Pfizer. The remaining authors declare that the research was conducted in the absence of any commercial or financial relationships that could be construed as a potential conflict of interest.

## Publisher’s Note

All claims expressed in this article are solely those of the authors and do not necessarily represent those of their affiliated organizations, or those of the publisher, the editors and the reviewers. Any product that may be evaluated in this article, or claim that may be made by its manufacturer, is not guaranteed or endorsed by the publisher.
